# Genome Sequences of 14 *Firmicutes* Strains Isolated from the Human Vagina

**DOI:** 10.1128/genomeA.00888-16

**Published:** 2016-09-29

**Authors:** Grace E. Deitzler, Maria J. Ruiz, Cory Weimer, SoEun Park, Lloyd Robinson, Kymberlie Hallsworth-Pepin, Aye Wollam, Makedonka Mitreva, Amanda L. Lewis, Warren G. Lewis

**Affiliations:** a Department of Molecular Microbiology, Washington University School of Medicine, St. Louis, Missouri, USA; b Department of Obstetrics and Gynecology, Washington University School of Medicine, St. Louis, Missouri, USA; c Department of Medicine, Washington University School of Medicine, St. Louis, Missouri, USA; d Center for Women’s Infectious Disease Research, Washington University School of Medicine, St. Louis, Missouri, USA; e McDonnell Genome Institute, Washington University School of Medicine, St. Louis, Missouri, USA

## Abstract

Research on vaginal infections is currently limited by a lack of available fully sequenced bacterial reference strains. Here, we present strains (now available through BEI Resources) and genome sequences for a set of 14 vaginal isolates from the phylum *Firmicutes*. These genome sequences provide a valuable resource for future research in understanding the role of Gram-positive bacteria in vaginal health and disease.

## GENOME ANNOUNCEMENT

A variety of health outcomes have been associated with specific bacteria or bacterial patterns colonizing the human vagina. For example, bacterial vaginosis (BV) is a common dysbiosis of the vaginal microbiota that is associated with increased risks of sexually transmitted infections and serious complications during pregnancy, such as intrauterine infections and preterm labor (1–6). BV is generally characterized by the absence of vaginal *Lactobacillus* and overgrowth of diverse microbes, including *Firmicutes*, such as *Anaerococcus, Finegoldia, Megasphaera, Peptoniphilus*, and *Veillonella* species. However, little is known about the strategies these bacteria use to colonize the vagina or whether they are involved in the causes and complications associated with BV. We isolated 15 *Firmicutes* from vaginal swabs of healthy and BV-affected pregnant and nonpregnant women. While some of these bacterial species may have sequenced reference strains, there are very few strains from the human vagina whose genomes have been previously sequenced.

Vaginal swabs were collected from nonpregnant and pregnant women according to Washington University institutional review board (IRB)-approved protocols 201108155 and 20110382, respectively. Organisms were isolated from these swabs anaerobically on solid medium. Isolation procedures and clinical information will be described in a future publication. The culture conditions for each strain have been provided to BEI Resources. Sequencing of 16s rRNA genes >1,400 bp were used to initially assign bacteria genus and species identifications by comparison with the NCBI ribosomal database. Genomic DNA was obtained using the Wizard genomic DNA purification kit (Promega).

*De novo* assembly of genomes were conducted using the One Button Velvet assembly pipeline (version 1.1.06) (7), with hash sizes of 31, 33, and 35 after downsizing the sample input data to 100× coverage. A minimum length for contigs was set (postassembly) at 200 bp. We performed a screen for core genes (as defined by the HMP [8]) on many of the assemblies to test for completeness of the genome. Gene annotation was performed using both *ab initio* and evidence-based (BLAST) predictions. Coding sequences were predicted using GeneMark and Glimmer3 (9, 10). Intergenic regions not identified by GeneMark and Glimmer3 were searched by BLAST in NCBI’s nonredundant bacterial (NR) database. The best prediction for each open reading frame was selected by evaluating all predictions against the best evidence (nonredundant bacterial, NR, and Pfam [11]) and resolving overlaps between adjacent coding genes. tRNA genes were determined using tRNAscan-SE (12) and noncoding RNA genes by RNAmmer (13) and Rfam (14). Metabolic pathways and subcellular localization were predicted using KEGG (15) and PSORTb (16), respectively, and functional domains were evaluated using InterProScan (17).

### Accession number(s).

These whole-genome shotgun projects have been deposited in GenBank under the accession numbers listed in Table 1. The sequences described in this paper are the first versions. We have also made the strains available to the research community by depositing them with the Biodefense and Emerging Infections (BEI) Research Resource Repository (see BEI numbers in Table 1).

**TABLE 1:** Strain names and accession numbers

Species	Strain	BEI catalog no.	Nucleotide sequence accession no.
*Anaerococcus tetradius*	MJR8151	HMS-1268	LRPM00000000
*Bacillus coagulans*	GED7749B	HMS-1281	LRPN00000000
*Clostridium perfringens*	MJR7757A	HMS-1290	LRPU00000000
*Enterococcus faecium*	MJR8396B	HMS-1267	LRPV00000000
*Finegoldia magna*	GED7760A	HMS-1285	LRPW00000000
*Megasphaera* sp.	MJR9396C	HMS-1269	LRVC00000000
*Peptoniphilus harei*	CMW7756A	HMS-1297	LRQE00000000
*Peptostreptococcus anaerobius*	MJR8628A	HMS-1263	LSQZ00000000
*Staphylococcus lugdunensis*	MJR7738	HMS-1293	LRQI00000000
*Staphylococcus simulans*	MJR7712	HMS-1283	LRQJ00000000
*Streptococcus mitis*	CMW7705B	HMS-1296	LRQR00000000
*Streptococcus pasteurianus*	GED7275A	HMS-1273	LSRA00000000
*Streptococcus salivarius*	GED7778A	HMS-1287	LRQS00000000
*Veillonella atypica*	CMW7756B	HMS-1301	LRQT00000000

## References

[1] Atashili J, Poole C, Ndumbe PM, Adimora AA, Smith JS 2008 Bacterial vaginosis and HIV acquisition: a meta-analysis of published studies. AIDS 22:1493–1501. doi:10.1097/QAD.0b013e3283021a37. 18614873 PMC2788489

[2] Hillier SL, Nugent RP, Eschenbach DA, Krohn MA, Gibbs RS, Martin DH, Cotch MF, Edelman R, Pastorek JG Jr, Rao AV 1995 Association between bacterial vaginosis and preterm delivery of a low-birth-weight infant. The Vaginal Infections and Prematurity Study Group. N Engl J Med 333:1737–1742. doi:10.1056/NEJM199512283332604. 7491137

[3] Hitti J, Hillier SL, Agnew KJ, Krohn MA, Reisner DP, Eschenbach DA 2001 Vaginal indicators of amniotic fluid infection in preterm labor. Obstet Gynecol 97:211–219. 11165584 10.1016/s0029-7844(00)01146-7

[4] Holst E, Goffeng AR, Andersch B 1994 Bacterial vaginosis and vaginal microorganisms in idiopathic premature labor and association with pregnancy outcome. J Clin Microbiol 32:176–186. 8126176 10.1128/jcm.32.1.176-186.1994 PMC262991

[5] Wiesenfeld HC, Hillier SL, Krohn MA, Landers DV, Sweet RL 2003 Bacterial vaginosis is a strong predictor of *Neisseria gonorrhoeae* and *Chlamydia trachomatis* infection. Clin Infect Dis 36:663–668. doi:10.1086/367658. 12594649

[6] Zhang X, Xu X, Li J, Li N, Yan T, Ju X 2002 Relationship between vaginal sialidase bacteria vaginosis and chorioammionitis. Zhonghua Fu Chan Ke Za Zhi 37:588–590. 12487930

[7] Zerbino DR, Birney E 2008 Velvet: algorithms for de novo short read assembly using de Bruijn graphs. Genome Res 18:821–829. doi:10.1101/gr.074492.107. 18349386 PMC2336801

[8] Human Microbiome Project Consortium 2012 A framework for human microbiome research. Nature 486:215–221. doi:10.1038/nature11209. 22699610 PMC3377744

[9] Borodovsky M, Mills R, Besemer J, Lomsadze A 2003 Prokaryotic gene prediction using GeneMark and GeneMark.Hmm. Curr Protoc Bioinformatics Chapter 4:Unit 4.5. doi:10.1002/0471250953.bi0405s01. 18428700

[10] Delcher AL, Harmon D, Kasif S, White O, Salzberg SL 1999 Improved microbial gene identification with GLIMMER. Nucleic Acids Res 27:4636–4641. doi:10.1093/nar/27.23.4636. 10556321 PMC148753

[11] Finn RD, Tate J, Mistry J, Coggill PC, Sammut SJ, Hotz HR, Ceric G, Forslund K, Eddy SR, Sonnhammer EL, Bateman A 2008 The Pfam protein families database. Nucleic Acids Res 36:D281–D288. doi:10.1093/nar/gkm960. 18039703 PMC2238907

[12] Lowe TM, Eddy SR 1997 tRNAscan-SE: a program for improved detection of transfer RNA genes in genomic sequence. Nucleic Acids Res 25:955–964. doi:10.1093/nar/25.5.0955. 9023104 PMC146525

[13] Lagesen K, Hallin P, Rødland EA, Staerfeldt H-H, Rognes T, Ussery DW 2007 RNAmmer: consistent and rapid annotation of ribosomal RNA genes. Nucleic Acids Res 35:3100–3108. doi:10.1093/nar/gkm160. 17452365 PMC1888812

[14] Griffiths-Jones S, Moxon S, Marshall M, Khanna A, Eddy SR, Bateman A 2005 Rfam: annotating non-coding RNAs in complete genomes. Nucleic Acids Res 33:D121–D124. doi:10.1093/nar/gki081. 15608160 PMC540035

[15] Kanehisa M, Goto S, Kawashima S, Okuno Y, Hattori M 2004 The KEGG resource for deciphering the genome. Nucleic Acids Res 32:D277–D280. doi:10.1093/nar/gkh063. 14681412 PMC308797

[16] Yu NY, Wagner JR, Laird MR, Melli G, Rey S, Lo R, Dao P, Sahinalp SC, Ester M, Foster LJ, Brinkman FS 2010 PSORTb 3.0: improved protein subcellular localization prediction with refined localization subcategories and predictive capabilities for all prokaryotes. Bioinformatics 26:1608–1615. doi:10.1093/bioinformatics/btq249. 20472543 PMC2887053

[17] Quevillon E, Silventoinen V, Pillai S, Harte N, Mulder N, Apweiler R, Lopez R 2005 InterProScan: protein domains identifier. Nucleic Acids Res 33:W116–W120. doi:10.1093/nar/gki442. 15980438 PMC1160203

